# Prevention of tumorigenesis in mice by exercise is dependent on strain background and timing relative to carcinogen exposure

**DOI:** 10.1038/srep43086

**Published:** 2017-02-22

**Authors:** Scott A. Kelly, Liyang Zhao, Kuo-Chen Jung, Kunjie Hua, David W. Threadgill, Yunjung Kim, Fernando Pardo Manuel de Villena, Daniel Pomp

**Affiliations:** 1 Department of Zoology, Ohio Wesleyan University, Delaware, Ohio 43015, USA; 2 Department of Genetics, University of North Carolina, Chapel Hill, North Carolina 27599, USA; 3 Department of Veterinary Pathobiology, College of Veterinary Medicine and Biomedical Sciences, Texas A&M University, College Station, Texas 77843, USA; 4 Department of Molecular and Cellular Medicine, College of Medicine, Texas A&M University, College Station, Texas 77843, USA

## Abstract

Among cancer diagnoses, colorectal cancer (CRC) is prevalent, with a lifetime risk of developing CRC being approximately 5%. Population variation surrounding the mean risk of developing CRCs has been associated with both inter-individual differences in genomic architecture and environmental exposures. Decreased risk of CRC has been associated with physical activity, but protective responses are variable. Here, we utilized a series of experiments to examine the effects of genetic background (strain), voluntary exercise (wheel running), and their interaction on azoxymethane (AOM)-induced intestinal tumor number and size in mice. Additionally, we investigated how the timing of exercise relative to AOM exposure, and amount of exercise, affected tumor number and size. Our results indicated that voluntary exercise significantly reduced tumor number in a strain dependent manner. Additionally, among strains where exercise reduced tumor number (A/J, CC0001/Unc) the timing of voluntary exercise relative to AOM exposure was crucial. Voluntary exercise prior to or during AOM treatment resulted in a significant reduction in tumor number, but exercise following AOM exposure had no effect. The results indicate that voluntary exercise should be used as a preventative measure to reduce risk for environmentally induced CRC with the realization that the extent of protection may depend on genetic background.

As with any complex disease, cancer risk is affected by both genetic and environmental factors. Of the 90% of colorectal cancers (CRC) not caused by inherited mutations in specific cancer-associated genes^1^,^2^, environmental variables are thought to contribute at least half of the risk for developing CRC^3^,^4^. Environmental and lifestyle contributions to cancer risk have been broadly categorized and include disparities in cigarette smoking, radiation exposure, stress, exposure to environmental pollutants, diet, obesity, and physical activity (refs 2 and 5 and references therein). Surprisingly, there is no evidence for interaction between CRC susceptibility alleles identified in genome-wide association studies and environmental factors like body mass index, alcohol, smoking, and diet^6^. However, the interaction between genetic risk factors and physical activity has not been investigated.

Consistent evidence exists for an inverse correlation between the risk for developing cancer and the level of physical activity for many cancer types^7^,^8^,^9^,^10^. A meta-analysis of 52 studies suggested that physical activity reduces the risk of developing CRC by 25%^11^. Most investigations into the relationship between physical activity and carcinogenesis involve very heterogeneous physical activity exposures that are broadly categorized as occupational or recreational (see ref. 5 and references therein), and, often, essential components (frequency, intensity, duration) of physical activity are not consistently measured. Moreover, when activity parameters are reported utilizing self-assessment recall surveys (often the only feasible method, see ref. 12) considerable measurement error may be introduced^13^,^14^. Alternatively, rodent models^15^ have been effectively utilized in controlled settings to investigate the relationship between forced or voluntary exercise and growth of transplantable, chemically induced, and/or spontaneous tumors (for a review see refs 16 and 17).

The effect of exercise on tumor growth inhibition in transplantable tumor systems^18^, or tumor occurrence reduction in chemically induced or spontaneous tumor models^19^, was especially striking in early studies using mice and rats. However, these studies frequently used exhaustive forced exercise protocols (i.e., 18 h of daily exercise in rotating drums, ref. 20) or high-dose inoculations of tumor cells, which limited extrapolation of the findings to humans. Recent mouse and rat studies utilizing more moderate forced and voluntary exercise and inoculation protocols have generally supported earlier findings, with the effect of exercise on cancer incidence and progression being more modest and variable^21^,^22^,^23^,^24^.

In this report, we investigated the effects of, and interactions between, genetics and voluntary physical activity on azoxymethane (AOM)-induced colon tumor number and size in mice. Additionally, we examined whether the timing of access to exercise relative to AOM exposure influenced carcinogenesis. We also examined exercise parameters (daily running distance, time spent running, average running speed, and maximum running speed) and tumor number and size, at the level of the individual, to elucidate dose response relationships. To identify potential molecular mechanisms we utilized transcriptional analysis of tumor and unaffected adjacent tissue.

## Results

### AOM 1–Strain by exercise interactions

**Figure 1:**
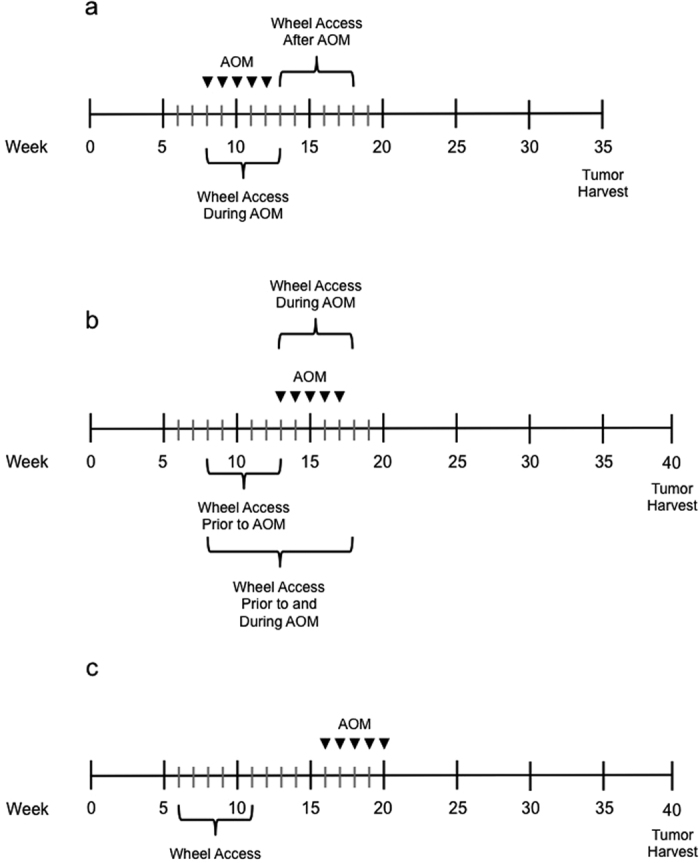
Experimental timeline for azoxymethane (AOM) treatment and voluntary running wheel access. (**a**) experiment 1 (*AOM 1*), 45 female mice from each of six strains (CC001/Unc, A/J, C57BL6/J, C58/J, KK/HIJ, I/LNJ). Mice were randomly assigned to one of the two-wheel access groups depicted or a third that never had access to running wheels. (**b**) experiments 2 and 3 (*AOM 2, 3*), for *AOM 2*, 96 (*n* = 48 males, *n* = 48 females) mice were utilized from the A/J strain. In *AOM 3*, 40 female A/J mice were used. Mice were randomly assigned to one of the three-wheel access groups depicted or a fourth that never had access to running wheels. (**c**) experiment 4 (*AOM 4*) 20 female A/J mice (*n* = 10, wheel access; *n* = 10, no wheel access).

We investigated the impact of genetic background, exercise, and their interaction on tumor number and size (Fig. 1). As a result of azoxymethane (AOM) administration there was considerable strain variability in mortality rate prior to tumor harvest at 35 weeks. Mortality numbers (as totals and percent of strain) were as follows: CC0001/Unc (*n* = 3, 6.7%), A/J (*n* = 4, 8.9%), C57BL6/J (*n* = 19, 42.2%), C58/J (*n* = 13, 28.9%), I/LNJ (*n* = 3, 6.7%), KK/HIJ (*n* = 11, 24.4%). The preceding individuals did not yield tumor data and were censured from analysis. Alternatively, some individuals were sacrificed prior to the 35-week endpoint due to declining health but still yielded data for tumor number and size. Age at early sacrifice ranged from 19–34 weeks and included individuals from the following strains: CC001/Unc (*n* = 5), C57BL6/J (*n* = 2), I/LNJ (*n* = 1), KK/HIJ (*n* = 2). A separate set of identical analyses excluding individuals sacrificed prior to 35 weeks yielded results very similar to those presented below. Cancer incidence among all individuals was 99.5% with only one I/LNJ mouse (access to wheels during AOM injections) not developing CRC. For descriptive statistics see Supplemental Tables 2 and 3.

When toxicity is observed with AOM, it is seen acutely due to disruption of the intestinal mucosa and consequential endotoxin effects on the liver. Animals that died acutely were excluded since their tumor response is unknown. Any animal euthanized early for a cancer endpoint (rectal prolapse for example) was included (as noted above). A near 100% tumor incidence in the control group is standard when using the AOM model in susceptible strains. The number of tumors is used as a surrogate for effect on incidence since each initiated tumor arises independent of all other tumors.

**Figure 2:**
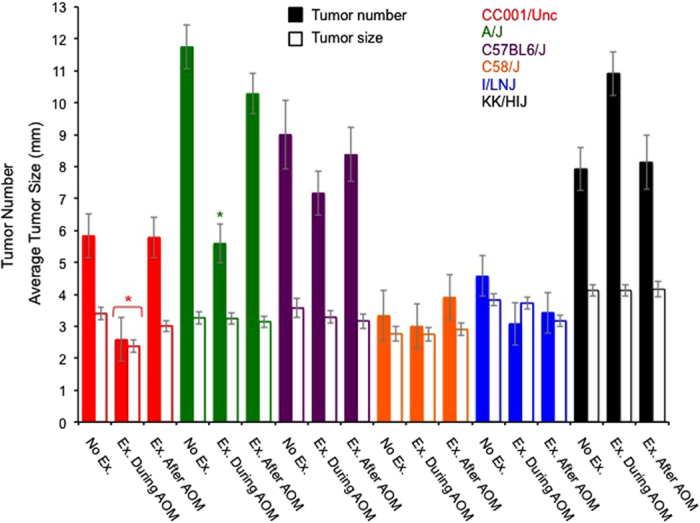
*AOM 1*–Effects of strain and exercise condition on tumor number and size (mm). General Linear Model revealed a statistically significant interaction between exercise condition [none, 5 weeks during azoxymethane (AOM) injections, 5 weeks following AOM injections] and strain on tumor number (*F*_10, 191_ = 6.051; *P* < 0.0001). Analyses of average tumor size (mm) indicated a statistically significant effect of strain (*F*_5, 189_ = 17.102; *P* < 0.0001), with no effect of exercise condition (*F*_2, 189_ = 2.584; *P* = 0.078). Asterisks denote significant effects of exercise condition within a strain. Bars represent estimated marginal means ± standard errors.

Full factorial analysis revealed a statistically significant exercise condition by strain interaction on tumor number (*F*_10, 191_ = 6.051; *P* < 0.0001). Analyses of average tumor size indicated a statistically significant effect of strain (*F*_5, 189_ = 17.102; *P* < 0.0001), with only a small effect of exercise condition (*F*_2, 189_ = 2.584; *P* = 0.078) and a non-significant interaction between exercise condition and strain (*F*_10, 189_ = 1.815; *P* = 0.060). For estimated marginal means and standard errors from the full factorial analysis see Fig. 2. Subsequently, we examined exercise condition effects within each strain. Despite a lack of significant interaction term for average tumor size, we performed additional analyses because ANOVAs typically have relatively low power to detect interactions^25^,^26^. Additionally, statistical significance for interactions is commonly judged at *P* = 0.1 rather than *P* = 0.05^27^.

For CC001/Unc exercise condition had a statistically significant effect on tumor number (*F*_2, 35_ = 7.992; *P* = 0.001) and average tumor size (*F*_2, 35_ = 6.277; *P* = 0.005). Post-hoc tests (Tukey HSD) revealed that exercise during the 5 weeks of AOM treatment significantly reduced tumor number and average size relative to other treatments (Fig. 2).

For A/J, exercise condition had a statistically significant effect on only tumor number (*F*_2, 38_ = 24.292; *P* < 0.0001). Post-hoc test (Tukey HSD) revealed that exercise during the 5 weeks of AOM treatment significantly reduced tumor number as compared to other treatments (Fig. 2).

For C57BL6/J, C58/J, I/LNJ, and KK/HIJ, exercise condition had no statistically significant effect on tumor number or average size. At the level of the individual, no wheel-running trait (distance run, time spent running, average running speed, or maximum running speed) explained a significant proportion of the variation in tumor number in either wheel access group for any of the strains.

### AOM 2 - Timing of exercise exposure

We next examined how the timing of exercise (relative to AOM treatment) influenced tumor number and size in both male and female A/J mice (Fig. 1b). Mortality prior to 40 weeks of age was observed in all groups except the group provided 5 weeks of wheel access coinciding with AOM treatment. Mortality numbers by group and sex were as follows: no access to a running wheels (*n* = 7, males), 5 weeks of access to a running wheel prior to AOM treatment (*n* = 3, males; *n* = 4, females), 10 weeks of wheel access spanning the 5 weeks prior and 5 weeks during AOM treatment (*n* = 3, males). The preceding individuals did not yield tumor data and were censured from analysis. Cancer incidence among all individuals was 100%. For descriptive statistics see Supplemental Table 4.

**Figure 3:**
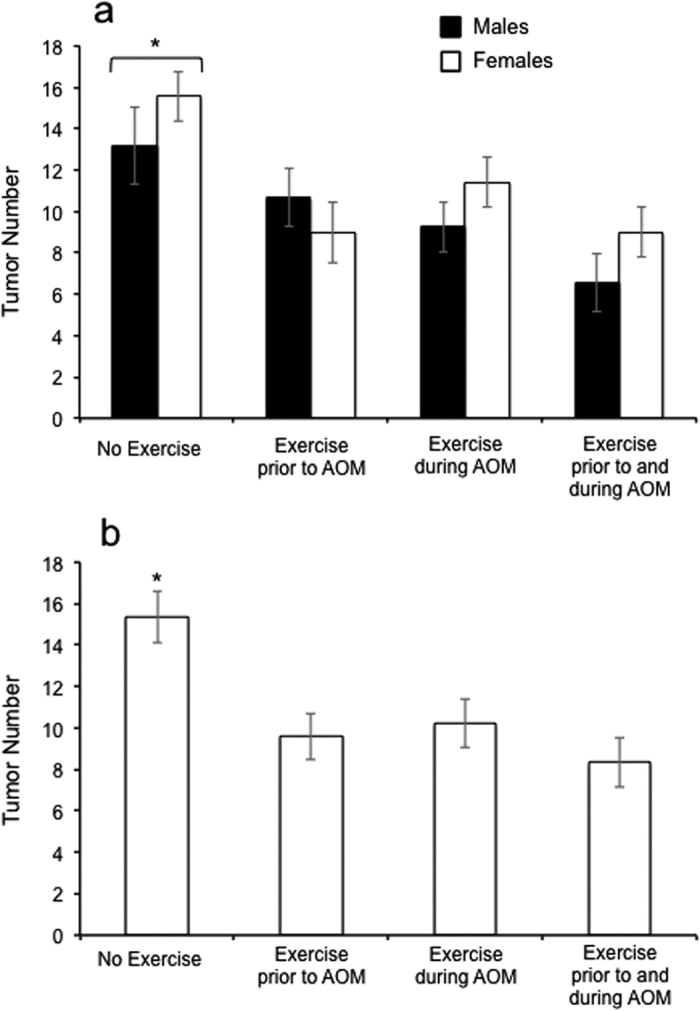
*AOM 2, 3*–Effects of sex and exercise condition on tumor number in A/J mice. Exercise treatments were: none, 5 weeks prior to azoxymethane (AOM) injections, 5 weeks during AOM injections, 10 weeks of wheel access spanning the 5 weeks prior and 5 weeks during AOM. For *AOM 2* (**a**), significant effect of exercise condition (*F*_3, 71_ = 7.039; *P* < 0.001), but not sex (*F*_1, 71_ = 1.835; *P* = 0.180) or the exercise condition-by-sex interaction (*F*_3, 71_ = 0.994; *P* = 0.401). For *AOM 3* (**b**), significant effect of exercise condition (*F*_3, 32_ = 6.355; *P* = 0.002), and post-hoc tests (Tukey HSD) between exercise conditions indicated that access to a running wheel at any time statistically significantly reduced tumor number relative to no exercise (*P* < 0.03, for all pairwise comparisons). Bars represent estimated marginal means ± standard errors.

Analysis of tumor number revealed a statistically significant effect of only exercise condition (*F*_3, 71_ = 7.039; *P* < 0.001) (Fig. 3a). Post-hoc tests (Tukey HSD) between exercise conditions indicated that access to a running wheel at any time showed a statistically significant reduction in tumor number relative to no exercise (*P* < 0.007, for all pairwise comparisons). However, there was no difference between tumor numbers of the three different exercise conditions (*P* > 0.238, for all pairwise comparisons). Analysis of tumor size revealed no statistically significant effect.

**Figure 4:**
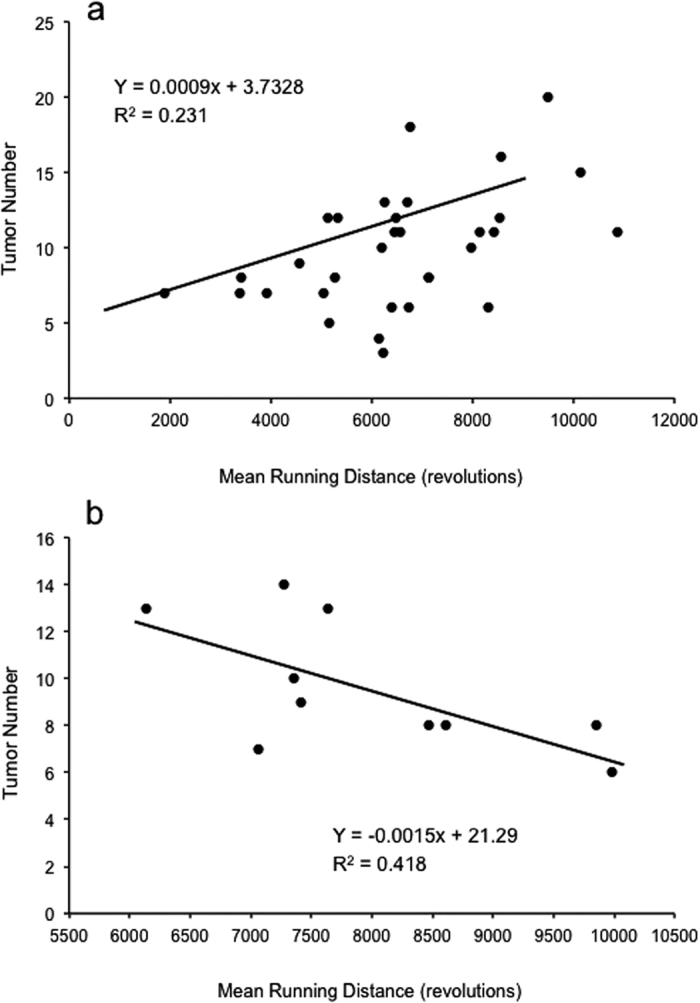
*AOM 2, 3*–Relationship among tumor number and mean wheel running distance during the final weeks of wheel access in female A/J mice. For *AOM 2* (**a**), regression analysis revealed mean wheel running distance explained a significant proportion of the variance in tumor number across all three wheel access groups [5 weeks prior to azoxymethane (AOM) injections, 5 weeks during AOM injections, 10 weeks of wheel access spanning the 5 weeks prior and 5 weeks during AOM] (*R* = 0.480, *R*^2^ = 0.231, *P* = 0.005). For *AOM 3* (**b**), relationship among tumor number and mean wheel running distance during the final weeks of wheel access in female A/J mice with 5 weeks of wheel access prior to azoxymethane (AOM) injections. Regression analysis revealed mean wheel running distance explained a significant proportion of the variance in tumor number (*R* = −0.646, *R*^2^ = 0.418, *P* = 0.044).

For males, at the level of individual, no wheel running trait explained a significant proportion of the variation in tumor number. For females, mean wheel running distance (Fig. 4a), speed, and maximum speed explained a significant proportion of the variance in tumor number across all three groups (*R* > 0.362, *R*^2^ > 0.131, *P* < 0.042). Additionally, mean time spent running explained a significant proportion of the variance in tumor number within groups that had access to wheels prior to (*R* = 0.751, *R*^2^ = 0.564, *P* = 0.032) and during (*R* = 0.666, *R*^2^ = 0.444, *P* = 0.018) AOM treatment.

### AOM 3–Timing of exercise exposure

We replicated *AOM 2* utilizing only females but changing the time of day for AOM administration (Fig. 1b). Mortality prior to 40 weeks of age was observed in all groups, albeit less than that observed in *AOM 2*, except for the group provided 5 weeks of wheel access prior to AOM treatment. Mortality numbers were as follows: no access to a running wheels (*n* = 2), 5 weeks of access to a running during AOM treatment (*n* = 1), 10 weeks of wheel access spanning the 5 weeks prior and 5 weeks during AOM treatment (*n* = 1). The preceding individuals did not yield tumor data and were censured from analysis. Cancer incidence among all individuals was 100%. For descriptive statistics see Supplemental Table 5.

Analysis of tumor number revealed a statistically significant effect of exercise condition (*F*_3, 32_ = 6.355; *P* = 0.002) (Fig. 3b). Post-hoc tests (Tukey HSD) between exercise conditions indicated that access to a running wheel at any time had a statistically significant reduction on tumor number relative to no exercise (*P* < 0.03, for all pairwise comparisons). Analysis of tumor size revealed no statistically significant effect of exercise condition.

At the level of individual across all wheel access groups combined, no wheel running trait explained a statistically significant proportion of the variance in tumor number. However, within the group that had access to wheels prior to the AOM treatment, mean wheel running distance explained a significant proportion of the variance in tumor number (*R* = −0.646, *R*^2^ = 0.418, *P* = 0.044) (Fig. 4b).

### AOM 4–Lasting effects of exercise

**Figure 5:**
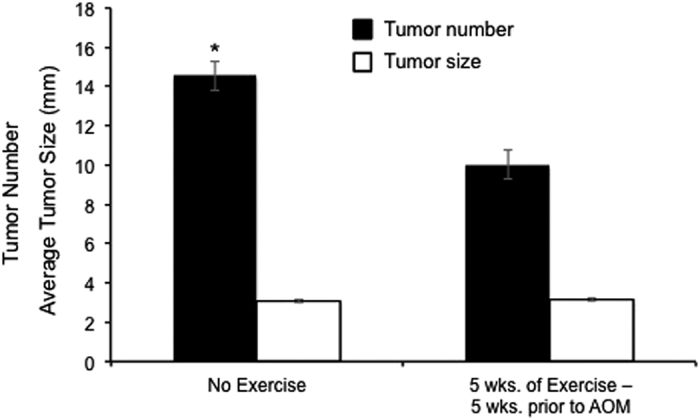
*AOM 4*–Effects of 5 weeks of running wheel exposure 5 weeks prior to AOM treatment (*n* = 19) *versus* no exposure (*n* = 18) in female A/J mice. Wheel access significantly reduce tumor number (*F*_1, 35_ = 18.524; *P* < 0.001), but not tumor size (*F*_1, 35_ = 0.138; *P* = 0.712). Bars represent estimated marginal means ± standard errors.

In the final experiment we examined if the protective effect of physical activity persists after individuals have stopped exercising (Fig. 1c). Mortality prior to 40 weeks of age was observed in both groups (no access to running wheels, *n* = 2; access to running wheels, *n* = 1), and these individuals did not yield tumor data. Cancer incidence among all individuals was 100%. For descriptive statistics see Supplemental Table 6. Analysis of tumor number revealed that 5 weeks of wheel access (5 weeks prior to AOM treatment) had a statistically significant reduction on tumor number (*F*_1, 35_ = 18.524; *P* < 0.001), but not size (Fig. 5). At the level of individual, the mean amount of wheel running (*R* = −0.572, *R*^2^ = 0.327, *P* = 0.010) and mean maximal running speed (*R* = −0.589, *R*^2^ = 0.347, *P* = 0.008) during the final week of wheel access each individually explained a significant proportion of the variance in tumor number (Supplemental Figure 1).

### Transcriptional analysis

**Figure 6:**
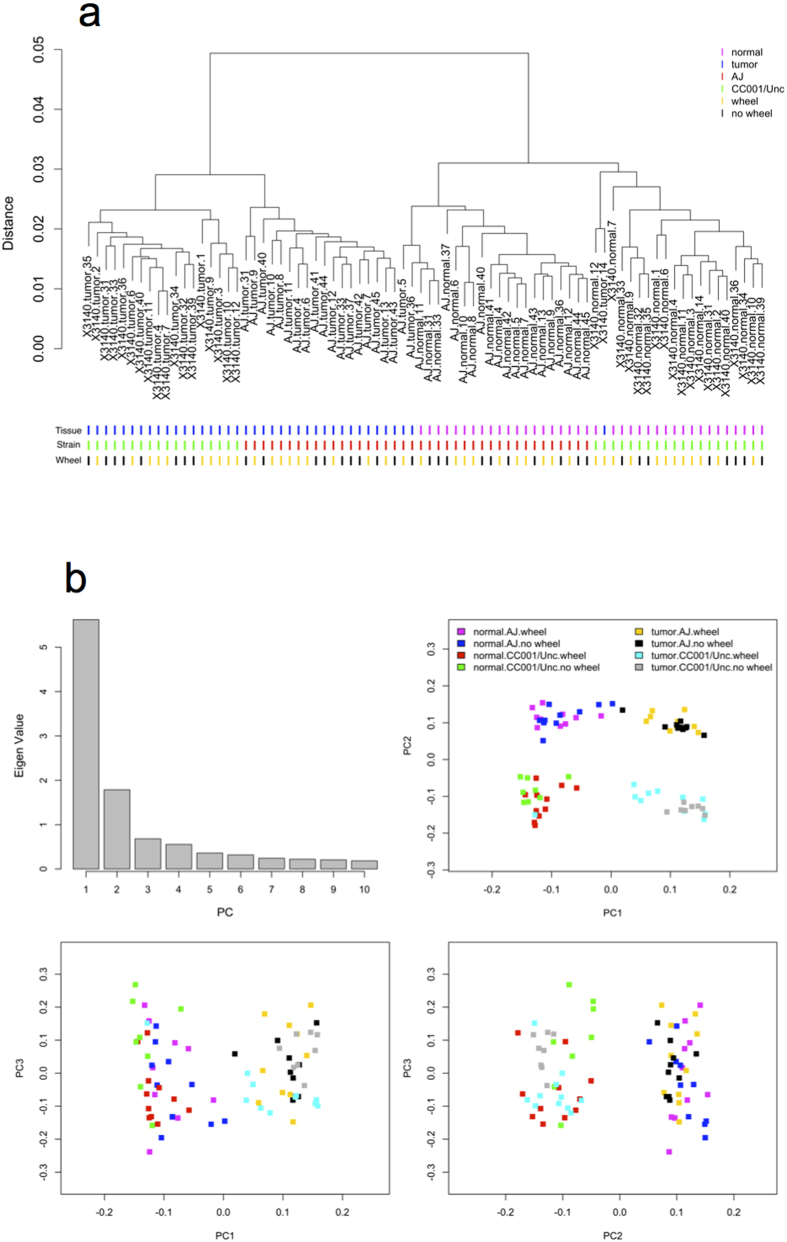
(**a**) Array-level, unsupervised descriptive summaries of gene expression using higherarchical clustering. (**b**) Eigenvalues and projection plots for the first three principal components (PC). Samples were from CC001/Unc and A/J mice provided no access to a running wheel for the duration of the experiment (no wheel) or 5 weeks of access to a running wheel coinciding with AOM treatment (wheel).

Hierarchical clustering and principal component analysis strongly indicate large expression differences by tissue followed by strain (Fig. 6). The first principal component (PC), which accounted for approximately 38% of the expression variance, was significantly associated with tissue differences between tumor and normal (ANOVA, *P* = 4.2E-32). PC2 accounted for approximately 12% of the expression variance and was associated with strain differences between A/J and CC001/Unc (ANOVA, *P* = 8.6E-46). In contrast to tissue and strain effects, wheel effects on gene expression were considerably smaller and marginally explained by PC3 (ANOVA, *P* = 0.05).

Differential expression analysis using all 77 samples at FDR 5% detected 20,119 differentially expressed transcripts for tumor vs. normal tissue, 4,833 transcripts for CC001/Unc vs. A/J, none for wheel vs. no wheel, 159 transcripts for tissue by wheel interaction, and none for strain by wheel interaction (Supplemental Table 7). These results are consistent with to hierarchical clustering and PCA plots, where tissue and strain dominated PC1 and PC2, respectively. Importantly, no differences were observed for wheel vs. no wheel even at FDR 30% emphasizing that the lack of a wheel effect is not due to lack of power. Supplemental Figure 2 shows the overlap of the differentially expressed genes (FDR < 0.3) with respect to wheel main effect, strain by wheel interaction effect, and tissue by wheel interaction effect and confirms that wheels have little to no effect on colon gene expression.

We performed functional enrichment analysis for the differentially expressed genes in Supplemental Table 7. Differentially expressed genes for tumor vs. normal at FDR q values (adjusted p-values) <0.05 were enriched for GO categories of cell cycle, DNA repair, mRNA processing, and other pathways related to cell survival. As expected, genes with strain effect at FDR q < 0.05 were enriched for diverse pathways, but did not form interpretable pathways. Enriched pathways for each factor of interests are listed in a Supplementary File 1 (GO_pathways).

To investigate whether the bulk of CC001/Unc vs. A/J strain effects are attributable to *cis* eQTL effects, we studied the impact of genetic sequence similarity on expression levels (Supplemental Table 8). Regardless of tissues, transcripts located in regions inherited from non-A/J CC founders were more likely to show differential expression between A/J and CC001/Unc genome-wide (*P* = 3.7E-35 for normal tissue; *P* = 3.3E-23 for tumor tissue). We repeated similar analysis for each chromosome individually. In Chromosomes 2, 8, 17, and 18, we observed an enrichment of transcripts showing strain effect among regions inherited from the other seven CC founders. This is expected because those chromosomes have a higher fraction of A/J inherited regions (Supplemental Figure 3) and hence higher power to detect the strain effect than the other chromosomes.

## Discussion

We have demonstrated that exercise significantly reduced tumorigenesis and the preventative effect was dependent on genetic background and the timing of physical activity relative to the exposure to the cancer-inducing agent (azoxymethane, AOM). Furthermore, we have shown that the protective effect of physical activity persists after individuals have stopped exercising. Importantly, we saw no evidence that physical activity was an effective therapeutic when exercise was initiated after completion of the exposure to AOM. We observed heterogeneity across experiments in the relationship between amount of exercise and the extent of tumor formation (i.e., the dose-response relationship). We discuss these findings in the context of human cohort studies and mouse model investigations into the association between physical activity and CRC risk and prognosis.

Literature examining CRCs and physical activity in humans has yielded variable conclusions as to the efficacy of exercise as a preventative measure for tumorigenesis. For example, Sanchez *et al*.^28^ concluded that one-hour of exercise per week lowered prevalence of polyps and adenomas in a multiethnic population. Conversely, Spence *et al*.^29^ systematically reviewed 20 cohort studies (11 that were “high-quality”) and concluded that evidence was not “sufficient to claim a *convincing* [sic] relationship exists between PA and CRC risk.” However, the authors acknowledge heterogeneity in the results of the reviewed studies–with 64% of the highest quality studies reporting at least one significant association between physical activity and CRC risk. Additionally, Spence *et al*.^29^ observed a non-significant trend across studies (*n* = 11) where cancer risk decreased as physical activity increased. It is worth noting that these dose-response relationship examinations are not true measures at the level of the individual (i.e., linear regression or correlational analyses), but rather a categorical comparison of multiple levels of ranges of physical activity (e.g., no activity, “low” activity, “high” activity). These types of broad physical activity exposure definitions (often out of necessity) may contribute to disparities between human cohort findings^30^.

We demonstrated exercise (an altered environment) reduces AOM-induced tumor formation in a strain dependent manner. Among the current strains, the one that was most susceptible in the absence of exercise, A/J, was also the most responsive to exercise as indicated by a nearly 50% reduction in tumor number. The response of KK/HIJ mice compared to A/J epitomizes the importance of gene-by-environment interactions associated with physical activity and cancer risk. When granted wheel access (and the opportunity to exercise) during AOM treatment, tumor number increased substantially in KK/HIJ mice albeit just short of significant (effects of exercise condition, *P* = 0.055). Exercise quantity (or type) could not explain the opposing effects in these two strains as wheel running distances (in revolutions per day) for both A/J and KK/HIJ mice were very similar (mean ± SE were 7274 ± 249 and 7314 ± 362, respectively). This discrepancy between strains in response to exercise may be indicative of the heterogeneity frequently observed between human cohort studies. And, the strain and the strain-by-exercise variation may potentially be mediated through immunological variation between strains^31^ and variation in immunoreactivity to wheel running^32^.

It is noteworthy that in *AOM 1* exercise reduced tumor number in a strain dependent manner only when the exercise occurred concurrent with AOM treatment. No strain showed a significant reduction in tumor number when exercise was administered following the AOM treatment. This conflicts with previous studies where pre and post inoculation exercise resulted in a marked protective effect^33^. In all our subsequent experiments AOM treatments were administered during or after wheel running, and we found remarkably consistent and highly repeatable results showing that exercise significantly reduced tumor number regardless of the timing. Our findings in A/J mice concurred with Colbert *et al*.^23^, who found negative energy balance induced by voluntary wheel running for 10 weeks significantly inhibited intestinal polyp formation in *Apc*^*Min*^ mice. Unique to the current experiments, we demonstrated that 5 weeks of exercise in the 5 weeks prior to AOM treatment resulted in a significant reduction in tumor number. This is an important first step to demonstrating the lasting effects of physical activity in CRC prevention, and may indicate that the positive effect of exercise on reducing cancer risk occurs during the carcinogenesis process, rather than on progression of initiated tumors.

Similar to previous findings in breast cancer murine models^34^, we found a significant negative relationship between mean running distance (during the final weeks of wheel access) and tumor number among individuals during *AOM 3* and *AOM 4*. However, in *AOM 2*, which was methodologically identical to *AOM 3* except for the timing of AOM exposure, we observed a significant positive relationship between tumor number and mean running distance. In an attempt to better understand the inconsistency found in *AOM 2*, we examined all weeks of wheel running data as well as the cumulative amount of running (revolutions summed across all days). The significant positive relationship between running distance and tumor number varied by group and week (see Fig. 1B for treatment schedule)–with the group with wheel access before AOM treatment revealing the most consistent positive relationships (weeks 2–5, and total wheel running across all weeks). There were no significant relationships observed within the group granted wheel access during AOM treatment. And, we only observed significant positive relationships during weeks 9 and 10 among the group with access to running wheels before and during AOM treatment. These inconsistencies may potentially be attributable to the overestimation of the duration of continuous running or high levels of inter-individual variability in “coasting” (not measured here but see ref. 35). Although there are differences between experiments at the level of the individual (i.e., correlation between exercise amount and tumor number), which require further investigation, across every experiment we clearly demonstrate that exercise (prior to or during AOM exposure) reduces tumor number at the ‘group’ level.

Based on our gene expression results we preliminarily conclude that the protective effects of wheel running on tumor multiplicity are not mediated through changes in the overall level of expression of genes in the colon. It is possible that the protective effects are due to transcriptional changes that are undetectable by microarray analysis (for example alternative splicing) or to posttranscriptional processes^36^. However, it is more likely that the benefits of exercise in CRC are mediated through systemic changes. For example, Pedersen *et al*.^32^ demonstrated that voluntary wheel running suppressed lung, liver, and skin tumor growth in C57BL/6 and the effects were mediated through epinephrine and IL-6 dependent NK cell mobilization.

In conclusion, our results indicate that voluntary exercise should be used as a preventative measure for colorectal cancer, with the realization that the extent of the protection may depend on genetic background and the timing of the activity relative to the onset of CRC. Our results also indicate that some exercise is better than none, but not necessarily that more exercise is consistently better than less. Nonetheless, these studies should be important in guiding future public health recommendations as this is the first demonstration that genetic background can interact with an environmental exposure (exercise) to modulate CRC risk.

## Materials and Methods

### Animals and carcinogen treatment

Mice were obtained from The Jackson Laboratory (A/J, C57BL6/J, C58/J, KK/HIJ, I/LNJ; selected for known susceptibility to CRC) or bred in house [CC001/Unc, a strain of the Collaborative Cross (CC) that had previously demonstrated high levels of voluntary wheel running^37^,^38^]. The CC is a multiparental recombinant inbred panel with A/J being one of the eight founder strains. Mice were housed by gender in groups of four in a viral free facility and maintained in the same room. At approximately eight-weeks of age individuals from each strain were randomly assigned to their respective treatment group. Mice assigned to exercise groups were individually housed during wheel exposure in a room dedicated to collecting activity measures, within the same facility. At the end of the 5 or 10 week wheel exposure mice were regrouped and housed in their original room. Mice were treated weekly for 5 weeks with intraperitoneal (IP) injections of azoxymethane (AOM, Sigma-Aldrich) at a dose of 10 mg/kg body weight (Fig. 1). This dose has been previously shown to maximize inter-strain differences while minimizing intra-strain variability^39^. AOM exposures were in the mornings for experiments *AOM 1, AOM 3*, and *AOM 4*, while exposure was in the afternoon for *AOM 2*. Following AOM treatment mice were weighed weekly and health status monitored continuously. At the conclusion of the experiments, mice were weighed, sacrificed, and colons removed from the cecum to the rectum and flushed with phosphate-buffered saline (PBS). Tumors were counted and maximum diameters measured utilizing a Leica MZ FL III microscope. This is the most widely used method for tumor size determination in the AOM model. Since the vast majority of tumors are polypoid adenomas, they are largely symmetrical so taking the longest axis provides an appropriate measure of relative tumor size. We did not histopathologically classify tumors, but previous pathology has shown that the AOM model predominantly induces adenomas that when given a promoter progress to carcinoma. Here, we focused on initiation and as such, used an adenoma endpoint. The AOM model is one of the most widely used models of sporadic colon cancer despite being largely adenomas.

Throughout all experiments mice were provided with water and food (Purina PMI pelleted rodent diet) *ad libitum*. All animal procedures were approved by and are in accordance with guidelines set forth by the Institutional Animal Care and Use Committee at The University of North Carolina at Chapel Hill.

### Exercise measurements

For individuals with access to running wheels, daily activity was monitored with Running Wheel Activity Software (AWM V9.2, Lafayette Instruments) via Activity Wheel Counters (model 86061, Lafayette Instruments) interfaced with computers. Wheel-running activity was recorded in 1-min intervals for 23–24 h each day of wheel access. From these recordings, the following daily traits were calculated: total daily revolutions, time spent running (i.e., cumulative 1-min intervals in which at least one revolution was recorded), average speed (total revolutions/time spent running), and maximum speed (highest number of revolutions in any 1-min interval within a 24 h period). We utilized mean values from the final weeks of exposure to wheels to examine relationships between amount/intensity of activity and tumor number and size at the level of the individual. Linear regression analysis was used to examine the relationship between running amount (total daily revolutions and time spent running) and intensity (average running speed, maximum running speed) and tumor number at the level of the individual.

### Experimental cohorts

#### AOM 1 - Strain by exercise interactions

Fifteen female mice from each of the six strains were used in one of three exercise conditions: no access to a running wheel for the duration of the experiment, 5 weeks of access to a running wheel coinciding with AOM treatment, or 5 weeks of wheel access following AOM treatment. Females were initially chosen because they typically run greater distances, run at higher average speeds, and for more minutes per day than males^40^. At sacrifice, a single tumor and unaffected adjacent colon tissue were harvested from each A/J and CC001/Unc mouse in the non-running wheel group and the group given concurrent access to running wheels during AOM exposure for transcriptional analysis. A General Linear Model (GLM) [Univariate GLM ANOVA (SPSS, Chicago, IL)] was utilized to examine the effects of the three exercise conditions and six strains as fixed effects on tumor number and size (mm). We also simultaneously examined interactions between exercise condition and strain. Distributions for tumor number and size were checked for normality prior to and following each analysis to determine if transformation was needed to stabilize variances among groups and/or improve normality of residuals. Statistical significance was judged at *P* < 0.05, and all *P*-values presented are two-tailed.

#### AOM 2 - Timing of exercise exposure

12 A/J males and 12 A/J females were randomly assigned to one of four exercise conditions: no access to a running wheel for the duration of the experiment, 5 weeks of access to a running wheel prior to AOM treatment, 5 weeks of wheel access during AOM treatment, or 10 weeks of wheel access spanning the 5 weeks prior and 5 weeks during AOM treatment. Males were included to test for any potential gender or gender by exercise effects. A GLM was utilized to examine the effects of the four exercise conditions and sex as fixed effects on tumor number and size (mm). We also simultaneously examined interactions between exercise condition and sex. Regressions were performed separately for each sex. Within a sex, regressions were performed across all groups and within each of the wheel access treatments.

#### AOM 3–Timing of exercise exposure

Because AOM injections in *AOM 1* were performed in the morning while those for *AOM 2* were performed in the afternoon and resulted in increased acute mortality, we replicated *AOM 2* but with morning AOM injections using 10 A/J females assigned to each of the same four treatment groups as described above. All other methods and analyses were the same as for *AOM 2*.

#### AOM 4–Lasting effects of exercise

Twenty A/J females were assigned to one of two exercise conditions: exposed to running wheels for 5 weeks, five weeks prior to AOM treatment or were never exposed to a running wheel. A GLM was utilized to examine the effects of exercise condition on tumor number and size (mm).

### Transcriptional analysis

#### Sample processing and quality control

Gene expression assays were conducted at the University of North Carolina Functional Genomics Core. RNA samples from a single tumor and unaffected adjacent colon tissue from each A/J and CC001/Unc mouse used in *AOM 1* were hybridized to an Affymetrix Mouse Gene 2.1 ST 96-Array plate. Array hybridization, washing, staining, and scanning were carried out using the Affymetrix GeneTitan system according to the manufacturer’s protocol. Following our prior reports^41^,^42^, we used the robust multiarray average method (RMA) implemented in the Affymetrix gene expression console (default settings, median polish and sketch-quantile normalization) to estimate normalized expression levels of transcripts. We excluded probes containing any SNPs in the A/J and CC001/Unc genomes^43^, control probesets, probesets without mRNA annotation, and lowly expressed probesets [maximum of log_2_ (expression level across samples) < 4]. To evaluate the overall performance of the arrays and identify outliers, we applied principal component analysis (PCA) and hierarchical clustering using the R function hclust with the average link function. The dataset for primary analyses consists of 30,953 probesets and 77 samples (20 A/J mice and 19 CC001/Unc mice) after careful quality control procedures on probe, probesets, and samples. The experimental design is shown in Supplemental Table 1. Samples were partitioned into 8 groups (2 × 2 × 2) for each combination of strain, wheel, and tissue. The sample sizes in each group ranges from 8 to 11.

#### Differential expression test

We used a linear mixed effect model to jointly analyze all 77 samples, while accounting for the correlation in adjacent tissues of each mouse. Let **y** be the expression of one probeset in *n* samples, **y** = (y_1_, y_1_, …, y_n_)^T^. The linear mixed effect model can be written as:


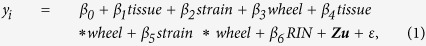


Fixed effects include tissue (tumor vs. normal), strain (CC001/Unc vs. A/J), wheel (wheel vs. no wheel), two-way interactions, and RNA integrity number (RIN). The random effect (***Zu***) includes a random intercept to account for dependencies between two tissues of each mouse. We also fitted linear fixed effect models within each tissue separately to study strain, wheel, and their interaction effects. The model is:


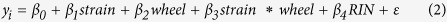


Within each strain, we used a linear mixed effect model to account for correlation between two tissues of each mouse. The model is:


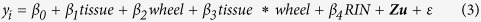


False discovery rate (FDR) correction was applied to transcript-based *P*-values to correct for multiple statistical comparisons (R package qvalue)^44^.

#### Functional clustering analysis

We used ConsensusPathDB^45^ to test differentially expressed genes for enrichment in Gene Ontology (GO) and canonical pathways from the Kyoto Encyclopedia of Genes and Genomes (KEGG), Reactome, WikiPathways, and MouseCyc^46^. For each of the functional categories, a hypergeometric test was performed to examine whether the overlap between our list of genes and those present in each reference category was higher than expected by chance.

#### Impact of genomic background on differential expression

We expect that transcripts located in genetic sequence dissimilarity regions between A/J and CC001/Unc are more likely to be differentially expressed between the two strains due to *cis* eQTL effects^42^. To test this hypothesis, we classified the genome of CC001/Unc into two categories (i.e., regions inherited from the A/J founder and regions inherited from the other seven CC founders). We also classified all the tested transcripts into two groups (i.e., differentially expressed transcripts or non-differentially expressed transcripts) for each factor of interest. Then, Fisher’s exact test was performed to test whether differentially expressed transcripts were enriched in regions inherited from the other seven CC founders.

## Additional Information

**How to cite this article**: Kelly, S. A. *et al*. Prevention of tumorigenesis in mice by exercise is dependent on strain background and timing relative to carcinogen exposure. *Sci. Rep.* **7**, 43086; doi: 10.1038/srep43086 (2017).

**Publisher's note:** Springer Nature remains neutral with regard to jurisdictional claims in published maps and institutional affiliations.

## Supplementary Material

Supplementary Dataset

Supplementary Material
