# Supplementary material for: Prevention of tumorigenesis in mice by exercise is dependent on strain background and timing relative to carcinogen exposure
Source: Sci Rep. 2017 Feb 22;7:43086. doi: 10.1038/srep43086 (PMC5320535; doi:10.1038/srep43086)
Supplement: Supplementary Material [file srep43086-s2.doc]

**Prevention of tumorigenesis in mice by exercise is dependent on strain background and timing relative to carcinogen exposure**

Scott A. Kelly^1, *, †^, Liyang Zhao^2, †^, Kuo-Chen Jung^2^, Kunjie Hua^2^, David W. Threadgill^3,4^, Yunjung Kim^2^, Fernando Pardo Manuel de Villena^2^, Daniel Pomp^2^

^1^Department of Zoology, Ohio Wesleyan University, Delaware, Ohio 43015, USA

^2^Department of Genetics, University of North Carolina, Chapel Hill, North Carolina 27599, USA

^3^Department of Veterinary Pathobiology, College of Veterinary Medicine and Biomedical Sciences, Texas A&M University, College Station, Texas 77843, USA

^4^Department of Molecular and Cellular Medicine, College of Medicine, Texas A&M University, College Station, Texas 77843, USA

^†^These authors contributed equally to this work.

**The following is supporting supplementary information.**

**Supplemental Table 1. Experimental design for gene expression assays from a single tumor and unaffected adjacent colon tissue from strains A/J and CC001/Unc. Samples were taken from individuals never granted wheel access (No wheel) and mice given 5 weeks of access to a running wheel coinciding with azoxymethane (AOM) treatment (Wheel).**

| **Strain** | **Wheel** | ***n*** | **Tumor tissue** | **Normal tissue** |
| --- | --- | --- | --- | --- |
| A/J | Wheel | 10 | 10 | 10 |
|  | No wheel | 10 | 10 | 10 |
| CC001/Unc | Wheel | 11 | 10 | 11 |
|  | No wheel | 8 | 8 | 8 |
| Total | | 39 | 38 | 39 |

**Supplemental Table 2. *AOM 1* - Descriptive statistics for tumor number for strains of mice never granted wheel access, granted 5 weeks of wheel access prior to azoxymethane (AOM) injections, or granted 5 weeks of wheel access during AOM injections.**

|  | N | Mean | SE | 95% CI  Lower | 95% CI  Upper | Range |
| --- | --- | --- | --- | --- | --- | --- |
|  | *No Wheel Access* | | | | | |
| CC001/Unc | 12 | 5.8 | 0.4 | 4.9 | 6.8 | 4-8 |
| A/J | 12 | 11.8 | 0.9 | 9.8 | 13.7 | 6-16 |
| C57BL6/J | 5 | 9.0 | 1.3 | 5.5 | 12.5 | 7-14 |
| C58/J | 9 | 3.3 | 0.2 | 2.8 | 3.9 | 2-4 |
| I/LNJ | 14 | 4.6 | 0.8 | 2.9 | 6.3 | 1-11 |
| KKHIJ | 13 | 7.9 | 0.9 | 6.0 | 9.9 | 2-14 |
|  | *Wheel Access – 5 weeks prior to AOM* | | | | | |
| CC001/Unc | 12 | 2.6 | 0.3 | 2.0 | 3.2 | 1-4 |
| A/J | 15 | 5.6 | 0.5 | 4.5 | 6.7 | 2-9 |
| C57BL6/J | 12 | 7.2 | 0.6 | 5.8 | 8.5 | 4-11 |
| C58/J | 11 | 3.0 | 0.6 | 1.7 | 4.3 | 1-8 |
| I/LNJ | 13 | 3.1 | 0.3 | 2.5 | 3.7 | 2-5 |
| KKHIJ | 12 | 10.9 | 1.1 | 8.5 | 13.3 | 4-17 |
|  | *Wheel Access – 5 weeks during AOM* | | | | | |
| CC001/Unc | 14 | 5.8 | 0.9 | 3.8 | 7.7 | 1-12 |
| A/J | 14 | 10.2 | 0.6 | 9.0 | 11.6 | 6-13 |
| C57BL6/J | 8 | 8.4 | 0.8 | 6.5 | 10.2 | 5-12 |
| C58/J | 11 | 3.9 | 0.5 | 2.7 | 5.1 | 2-8 |
| I/LNJ | 14 | 3.4 | 0.6 | 2.2 | 4.7 | 0-9 |
| KKHIJ | 8 | 8.1 | 0.6 | 6.7 | 9.6 | 6-11 |

**Supplemental Table 3. *AOM 1* - Descriptive statistics for tumor size (mm) for strains of mice never granted wheel access, 5 weeks of wheel access prior to azoxymethane (AOM) injections, or 5 weeks of wheel access during AOM injections.**

|  | N | Mean | SE | 95% CI  Lower | 95% CI  Upper | Range |
| --- | --- | --- | --- | --- | --- | --- |
|  | *No Wheel Access* | | | | | |
| CC001/Unc | 12 | 3.4 | 0.25 | 2.9 | 4.0 | 2.3-5.5 |
| A/J | 12 | 3.3 | 0.13 | 3.0 | 3.6 | 2.6-4.0 |
| C57BL6/J | 5 | 3.6 | 0.42 | 2.4 | 4.7 | 2.8-4.8 |
| C58/J | 9 | 2.8 | 0.19 | 2.3 | 3.2 | 1.6-3.4 |
| I/LNJ | 14 | 3.8 | 0.22 | 3.4 | 4.3 | 1.5-4.9 |
| KKHIJ | 13 | 4.1 | 0.21 | 3.7 | 4.6 | 3.1-5.4 |
|  | *Wheel Access – 5 weeks prior to AOM* | | | | | |
| CC001/Unc | 12 | 2.4 | 0.14 | 2.1 | 2.7 | 1.6-3.1 |
| A/J | 15 | 3.2 | 0.11 | 3.0 | 3.5 | 2.4-4.0 |
| C57BL6/J | 12 | 3.3 | 0.18 | 2.9 | 3.7 | 2.4-4.8 |
| C58/J | 10 | 2.8 | 0.20 | 2.3 | 3.2 | 1.9-4.0 |
| I/LNJ | 13 | 3.7 | 0.21 | 3.3 | 4.2 | 2.7-5.4 |
| KKHIJ | 12 | 4.1 | 0.21 | 3.7 | 4.6 | 3.1-5.9 |
|  | *Wheel Access – 5 weeks during AOM* | | | | | |
| CC001/Unc | 14 | 3.0 | 0.20 | 2.6 | 3.4 | 2.0-5.0 |
| A/J | 14 | 3.1 | 0.09 | 2.9 | 3.3 | 2.5-3.8 |
| C57BL6/J | 8 | 3.2 | 0.21 | 2.7 | 3.7 | 2.3-4.2 |
| C58/J | 11 | 2.9 | 0.21 | 2.4 | 3.4 | 2.0-4.2 |
| I/LNJ | 13 | 3.2 | 0.19 | 2.8 | 3.6 | 2.5-4.5 |
| KKHIJ | 8 | 4.1 | 0.29 | 3.4 | 4.8 | 2.4-4.7 |

**Supplemental Table 4. *AOM 2* - Descriptive statistics for tumor number and size (mm) for A/J mice never granted wheel access, granted 5 weeks of wheel access prior to azoxymethane (AOM) injections, granted 5 weeks of wheel access during AOM injections, or granted 10 weeks of wheel access spanning the 5 weeks prior and 5 weeks during AOM injections.**

|  | Sex | N | Mean | SE | 95% CI  Lower | 95% CI  Upper | Range | N | Mean | SE | 95% CI  Lower | 95% CI  Upper | Range |
| --- | --- | --- | --- | --- | --- | --- | --- | --- | --- | --- | --- | --- | --- |
|  |  | *No Wheel Access* | | | | | | *Wheel Access – 5 weeks prior to AOM* | | | | | |
| Tumor Number | Male | 5 | 13.2 | 2.3 | 6.8 | 19.6 | 8-20 | 9 | 10.7 | 1.3 | 7.6 | 13.8 | 6-18 |
| Tumor Number | Female | 12 | 15.6 | 1.5 | 12.2 | 18.9 | 7-26 | 8 | 9.0 | 1.4 | 5.6 | 12.4 | 3-15 |
| Tumor Size (mm) | Male | 5 | 2.3 | 0.19 | 1.8 | 2.8 | 1.8-2.9 | 9 | 2.0 | 0.21 | 1.5 | 2.5 | 1.0-2.9 |
| Tumor Size (mm) | Female | 12 | 1.8 | 0.10 | 1.6 | 2.0 | 1.3-2.4 | 8 | 2.1 | 0.13 | 1.8 | 2.4 | 1.4-2.7 |
|  |  | *Wheel Access – 5 weeks during AOM* | | | | | | *Wheel Access – 10 weeks, prior to and during AOM* | | | | | |
| Tumor Number | Male | 12 | 9.3 | 1.3 | 6.5 | 12.0 | 2-16 | 9 | 6.6 | 0.9 | 4.3 | 8.7 | 4-12 |
| Tumor Number | Female | 12 | 11.4 | 1.3 | 8.6 | 14.2 | 6-20 | 12 | 9.0 | 0.9 | 7.0 | 11.0 | 5-16 |
| Tumor Size (mm) | Male | 12 | 2.0 | 0.15 | 1.7 | 2.4 | 1.2-3.1 | 9 | 2.1 | 0.16 | 1.7 | 2.5 | 1.3-2.9 |
| Tumor Size (mm) | Female | 12 | 1.9 | 0.08 | 1.7 | 2.1 | 1.5-2.4 | 12 | 2.1 | 0.08 | 1.9 | 2.2 | 1.6-2.6 |

**Supplemental Table 5. *AOM 3* - Descriptive statistics for tumor number and size (mm) for female A/J mice never granted wheel access, granted 5 weeks of wheel access prior to azoxymethane (AOM) injections, granted 5 weeks of wheel access during AOM injections, or 10 weeks of wheel access spanning the 5 weeks prior and 5 weeks during AOM injections.**

|  | N | Mean | SE | 95% CI  Lower | 95% CI  Upper | Range | N | Mean | SE | 95% CI  Lower | 95% CI  Upper | Range |
| --- | --- | --- | --- | --- | --- | --- | --- | --- | --- | --- | --- | --- |
|  | *No Wheel Access* | | | | | | *Wheel Access – 5 weeks prior to AOM* | | | | | |
| Tumor Number | 8 | 15.3 | 1.4 | 12.1 | 18.7 | 10-21 | 10 | 9.6 | 0.9 | 7.6 | 11.6 | 6-14 |
| Tumor Size (mm) | 8 | 3.0 | 0.08 | 2.8 | 3.2 | 2.6-3.2 | 10 | 3.1 | 0.08 | 2.9 | 3.3 | 2.7-3.4 |
|  | *Wheel Access – 5 weeks during AOM* | | | | | | *Wheel Access – 10 weeks, prior to and during AOM* | | | | | |
| Tumor Number | 9 | 10.2 | 1.4 | 7.0 | 13.5 | 5-17 | 9 | 8.3 | 1.0 | 5.9 | 10.7 | 5-13 |
| Tumor Size (mm) | 9 | 2.8 | 0.17 | 2.8 | 3.2 | 2.2-3.5 | 9 | 2.9 | 0.14 | 2.6 | 3.2 | 2.2-3.3 |

**Supplemental Table 6. *AOM 4* - Descriptive statistics for tumor number and size (mm) for female A/J mice never granted wheel access and mice granted 5 weeks of wheel access 5 weeks prior to AOM treatment.**

|  | **No Wheel Access** | | | | | | **Wheel Access** | | | | | |
| --- | --- | --- | --- | --- | --- | --- | --- | --- | --- | --- | --- | --- |
|  | N | Mean | SE | 95% CI  Lower | 95% CI  Upper | Range | N | Mean | SE | 95% CI  Lower | 95% CI  Upper | Range |
| Tumor Number | 18 | 14.6 | 0.8 | 12.9 | 16.2 | 8-21 | 19 | 10.0 | 0.7 | 8.5 | 11.5 | 5-17 |
| Tumor Size (mm) | 18 | 3.1 | 0.07 | 2.9 | 3.2 | 2.7-3.7 | 19 | 3.1 | 0.12 | 2.9 | 3.4 | 2.3-4.3 |

**Supplemental Table 7.** Differential expression results for gene expression assays from a single tumor and unaffected (Normal) adjacent colon tissue from strains A/J and CC001/Unc. Samples were taken from individuals never granted wheel access (No wheel) and mice given 5 weeks of access to a running wheel coinciding with azoxymethane (AOM) treatment (Wheel).

| **Analysis** | **Contrast**^a^ | **FDR** | **# of Differentially**  **Expressed Transcripts** | **# of**  **UP-regulated** | **# of**  **DOWN-regulated** |
| --- | --- | --- | --- | --- | --- |
| All samples | Tumor vs. Normal | 0.05 | 20 119 | 10 108 | 10 011 |
|  | CC001/Unc vs. A/J | 0.05 | 4833 | 2562 | 2271 |
|  | Wheel vs. No wheel | 0.05 (0.3) | 0 (0) | 0 (0) | 0 (0) |
|  | Tissue * Wheel | 0.05 (0.3) | 159 (1331) | 33 (590) | 126 (741) |
|  | Strain * Wheel | 0.05 (0.3) | 0 (0) | 0 (0) | 0 (0) |
| Within tumor | CC001/Unc vs. A/J | 0.05 | 3206 | 1681 | 1525 |
|  | Wheel vs. No wheel | 0.05 (0.3) | 1 (24) | 1 (20) | 0 (4) |
|  | Strain * Wheel | 0.05 (0.3) | 3 (1761) | 0 (526) | 3 (1235) |
| Within normal | CC001/Unc vs. A/J | 0.05 | 2370 | 1263 | 1107 |
|  | Wheel vs. No wheel | 0.05 (0.3) | 4 (48) | 4 (19) | 0 (29) |
|  | Strain * Wheel | 0.05 (0.3) | 0 (164) | 0 (80) | 0 (84) |
| Within CC001/Unc | Tumor vs. Normal | 0.05 | 16 753 | 8723 | 8030 |
|  | Wheel vs. No wheel | 0.05 (0.3) | 0 (0) | 0 (0) | 0 (0) |
|  | Tissue * Wheel | 0.05 (0.3) | 0 (106) | 0 (58) | 0 (48) |
| Within A/J | Tumor vs. Normal | 0.05 | 15 859 | 8275 | 7584 |
|  | Wheel vs. No wheel | 0.05 (0.3) | 0 (0) | 0 (0) | 0 (0) |
|  | Tissue * Wheel | 0.05 (0.3) | 0 (0) | 0 (0) | 0 (0) |

^a^Interaction terms are represented with * separating grouping variables.

**Supplemental Table 8.** The impact of genetic background on differential gene expression for assays from a single tumor and unaffected (Normal) adjacent colon tissue from strains A/J and CC001/Unc.

| **Chromosome** | **A/J region**  **in CC001/Unc (%)^a^** | **Normal tissue**  **(*P*-value)^b^** | **Tumor tissue**  **(*P*-value)^b^** |
| --- | --- | --- | --- |
| Whole genome | 19.1% | **3.7E-35** | **3.3E-23** |
| Chr1 | 7.4% | **0.0027** | 0.18 |
| Chr2 | 77.6% | **7.4E-5** | **3.5E-5** |
| Chr3 | 8.0% | 0.62 | **0.01** |
| Chr4 | 0% | 0.77 | 0.96 |
| Chr5 | 3.8% | 0.52 | 0.78 |
| Chr6 | 38.6% | 0.27 | 0.53 |
| Chr7 | 0% | 0.79 | 0.73 |
| Chr8 | 79.6% | **0.05** | **0.004** |
| Chr9 | 0% | 0.98 | 0.81 |
| Chr10 | 0% | 0.95 | 0.90 |
| Chr11 | 1.2% | 0.71 | 0.27 |
| Chr12 | 0% | 0.89 | 0.99 |
| Chr13 | 10.9% | 0.18 | 0.31 |
| Chr14 | 0% | 0.56 | 0.96 |
| Chr15 | 0% | 0.97 | 0.65 |
| Chr16 | 0% | 0.80 | 0.76 |
| Chr17 | 45.2% | **1.0E-5** | **0.01** |
| Chr18 | 51.4% | **4.1E-4** | **0.001** |
| Chr19 | 45.1% | 0.12 | 0.57 |
| ChrX | 0% | 0.83 | 0.81 |

^a^percentage of regions in CC001/Unc haplotype that were inherited from A/J. Such regions are also indicated by yellow lines in Supplemental Figure 3.

^b^*P*-value is based on Fisher’s exact test. For each tissue, Fisher’s exact test was performed to test whether differentially expressed transcripts between A/J and CC001/Unc were enriched in regions inherited from the other seven CC founders.

**
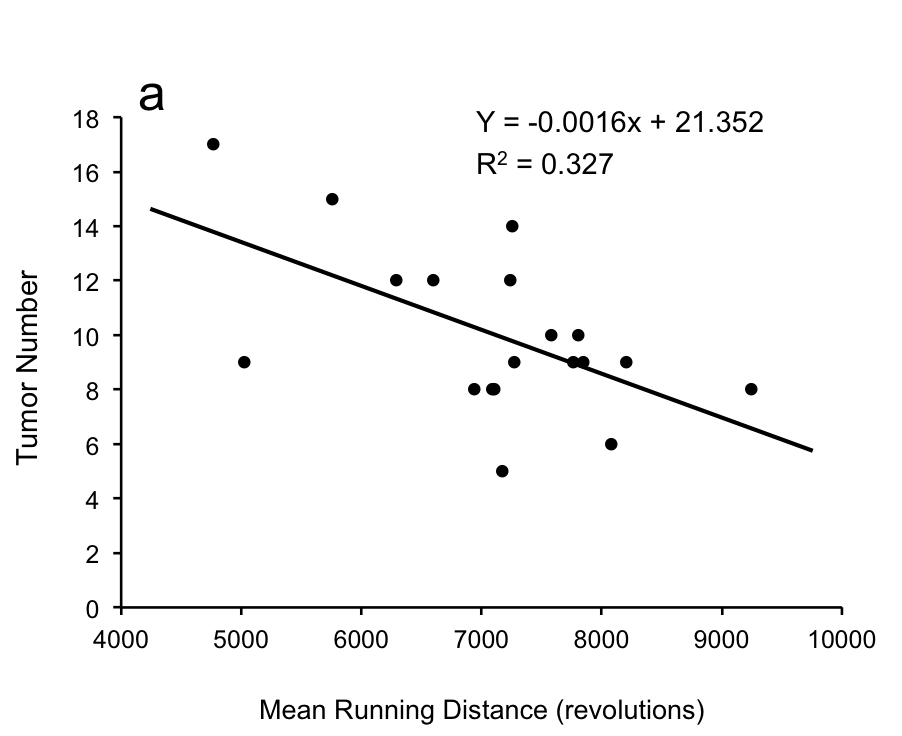
**

**
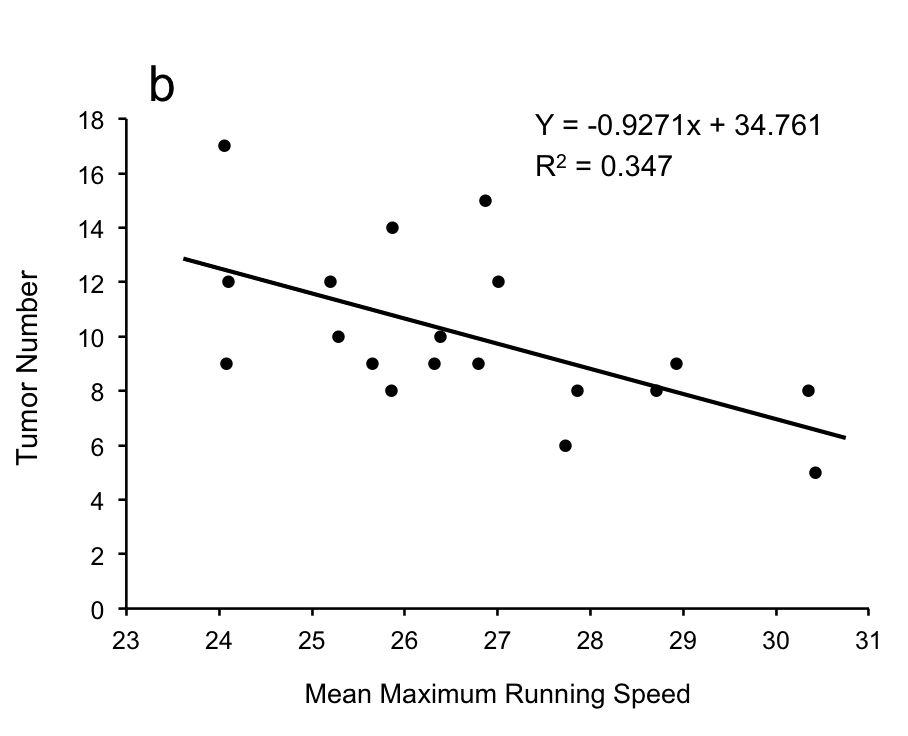
**

**Supplemental Figure 1.** *AOM4* – Relationship among tumor number and mean wheel running distance (**A**) and mean maximal running speed (**B**) during the final weeks of wheel access in female A/J mice with 5 weeks of wheel access 5 weeks prior to azoxymethane (AOM) injections. Regression analyses revealed mean amount of wheel running (*R* = -0.572, *R^2^* = 0.327, *P* = 0.010) and mean maximal running speed (*R* = -0.589, *R^2^* = 0.347, *P* = 0.008) each explained a significant proportion of the variance in tumor number.


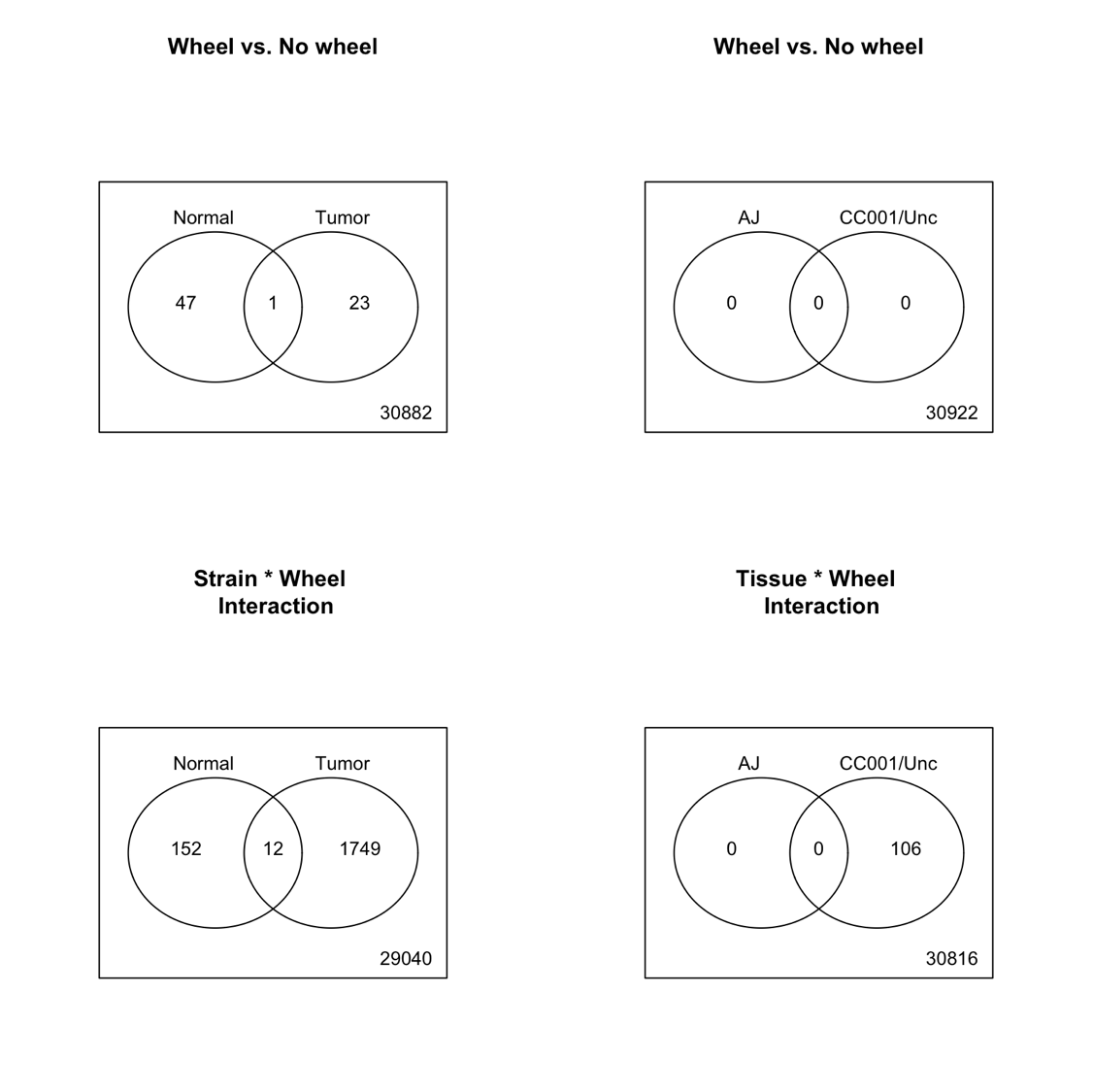


**Supplemental Figure 2.** Venn diagram showing the overlap of the differentially expressed genes (FDR < 0.3) with respect to wheel main effect, strain-by-wheel interaction effect, and tissue-by-wheel interaction effect. Samples were from CC001/Unc and A/J mice provided no access to a running wheel for the duration of the experiment (no wheel) or 5 weeks of access to a running wheel coinciding with AOM treatment (wheel). The number in lower right corner of each Venn diagram refers to the number of tested probe sets that do not belong to any category.


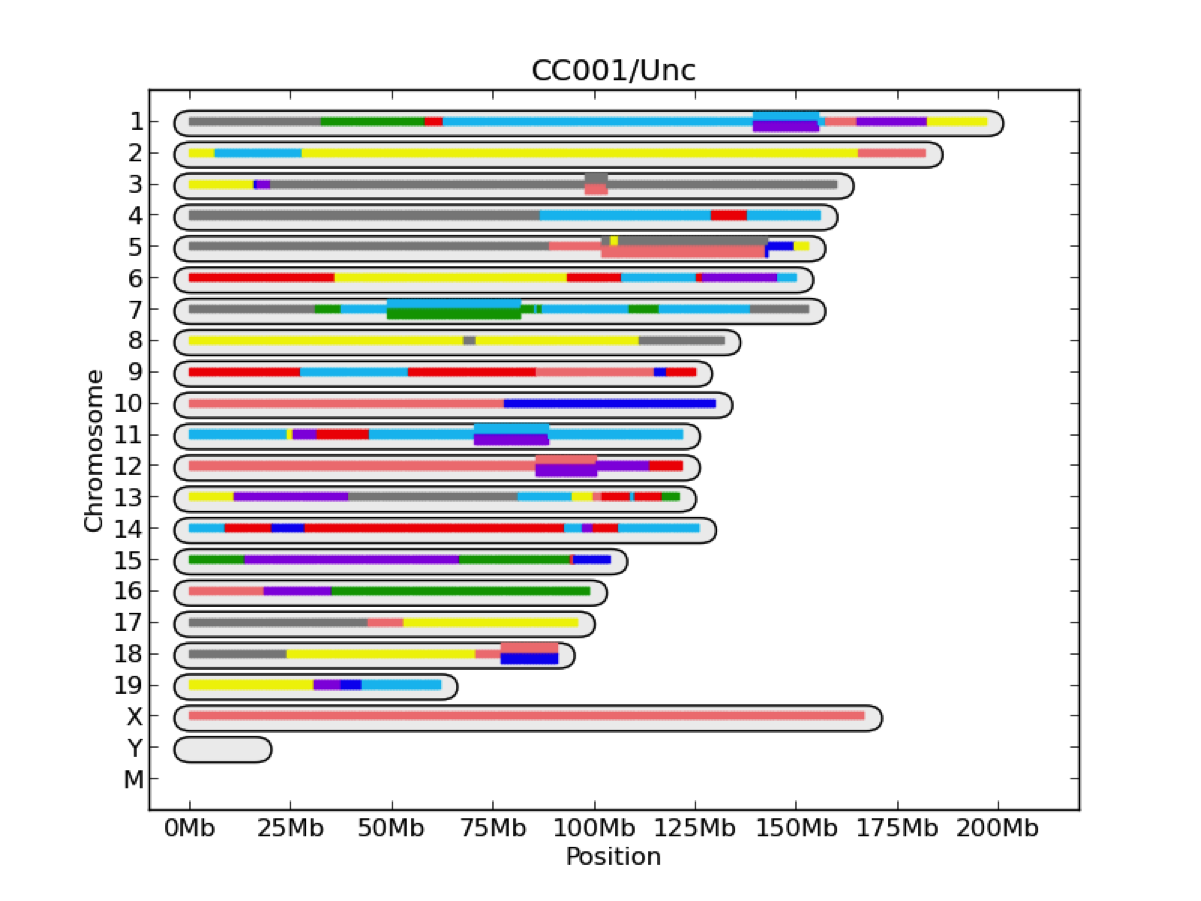


**Supplemental Figure 3.**  CC001/Unc haplotype. In each chromosome, regions inherited from A/J are indicated by yellow lines.
